# Supplementary material for: The Dual Prey-Inactivation Strategy of Spiders—In-Depth Venomic Analysis of Cupiennius salei
Source: Toxins (Basel). 2019 Mar 19;11(3):167. doi: 10.3390/toxins11030167 (PMC6468893; doi:10.3390/toxins11030167)
Supplement: Supplementary file 1 [file toxins-11-00167-s001.zip › Supplementary Fig S1.1-S1.19/Supplementary Fig S1.5.pdf]

Supplementary Fig S1.5

CLUSTAL O(1.2.4) multiple sequence alignment: Carboxypeptidases A-like

```
TR_A0A087UBY6_STEMI      MISFPSYPLIISCLMVAWVLPLTQTTDIDFVYHNYQSMTSVLHQLANEYPQLTKLYSIGK
CPA_VIRFA                -MSPPSLRLL-APLFLLSLAWVCSSVDVPFSYHDHPSMTRLLRIINQENPELTRLYSVGK
CPA_CUPSA                ------MFPS-VLLLLLLCAGLSSCVDVPFSYHDFSSMSRLMHLLHDENPELTRIYSVGK
CPA_ALOMA                ------MLPL---VFLLAVTGGALCLDIPFSYHDYSSMTRVLHDLHEEYPRMTSLYTVGK
                                      :::          *: * **:. **: ::: : :* *.:* :*::**

TR_A0A087UBY6_STEMI      STQGRELWVILVTSDPQEEPLLKPNVKYVANMHGNEAVGRELMLHLTSYLLKNYGKDDYV
CPA_VIRFA                STQGRELWVLLVTHEPDHEPILKPNVKYIANMHGNEAVGREMLLQLASYLVSGYGQDQYV
CPA_CUPSA                STQGRDLWVLLITHDPDSEPTLKPNVKYIANMHGNEAVGREMLLHLASYLVSAYGSDDYV
CPA_ALOMA                SGQGRELWVLLITREPESEPTLKPNVKYVANMHGNEAVGREMLLHLADHLLSSYGHDEYV
                         * ***:***:*:* :*: ** *******:************::*:*:.:*:. ** *:**

TR_A0A087UBY6_STEMI      TWLMDNTRIHIMPSMNPDGFEASVEGQCSGVQGRNNANSLDLNRNFPDYFAGNPEFPQAE
CPA_VIRFA                TWLLDNTRVHLMPSMNPDGFEASSEGECSGTHGRGNANSFDLNRNFPDYFTGDQVMIQPE
CPA_CUPSA                TWLLDHTRVHLMPSMNPDGFEASTEGQCSGTQGRGNTNSFDLNRNFPDYFTGDQDKLQPE
CPA_ALOMA                TWLLDNTRVHLMPSMNPDGFEASTEGQCSGTQGRGNSQSFDLNRNFPDYFTGEQDRMQPE
                         ***:*:**:*:************ **:***.:**.*::*:**********:*:    * *

TR_A0A087UBY6_STEMI      TLAVIDWLKRTQFVLSGNLHGGALVASYPFDNIPANSPKGFRNFGSPSMTPDDDVFRHLA
CPA_VIRFA                TRAIMNWMRQTQFVLSANLHGGALVASYPFDNIPQNTRKGFRTIGSPSATPDDDVFRHLA
CPA_CUPSA                TRAVMRWMAQTQFVLSANLHGGALVASYPFDNVPPNTPKGFRSSHSPSRTPDDDVFRHLA
CPA_ALOMA                TRSVMRWMASTQFVLSANLHGGALVASYPFDNVPPNTPKGFRSAHSPSLTPDDDVFRHLA
                         * ::: *:  ******.***************:* *: ****.  *** ***********

TR_A0A087UBY6_STEMI      QSYSFNHKNMYLEVTCRDGSPPFTNGTTNGAAWYALKGGMQDYNYVWAGCMEITLEISCC
CPA_VIRFA                ETYSFNHKNMYVGSSCRDGTPEFANGTTNGAAWYPLKGGMQDYNYVWGGCMEITLEISCC
CPA_CUPSA                ETYSFNHKSMYAGVSCRDGTPEFLNGTTNGAAWYPLKGGMQDYNYVWAGCMEITLEISCC
CPA_ALOMA                QTYSFSHKNMYAGASCRDGTPEFQNGTTNGAAWYPLKGGMQDYNYVWAGCMEITLEISCC
                         ::***.**.**   :****:* * ********** ************.************

TR_A0A087UBY6_STEMI      KYPKYHQLPKFWADNQKALLQYLGEAHQGVWGQVTDPDKSPVFNASIVVKGRKFGSRTTR
CPA_VIRFA                KYPMYHRLPQFWADNKHALLKYLGEAHQGVWGVVSDSNNNPLQNASIKVKGRKFGSKTTT
CPA_CUPSA                KFPRAHRLPQFWEDNKHALLKYLGEAHQGVWGVVSDSNNNPLNNVSIKVAGRQFGSRTTH
CPA_ALOMA                KYPRAHRLPQFWEDNRLALLKYLGEAHQGVWGVVTDANNNPLRNVSIKIQGRQFGSRTTP
                         *:*  *:**:** **: ***:*********** *:* ::.*: *.** : **:***:**

TR_A0A087UBY6_STEMI      RGEYWRILRPGTYTIQAQAEGYDPVEVTFPVAKGVTIQNITLYPQASTYYEPRSDYTSMR
CPA_VIRFA                RGEYWRILRPGTYNIEVAADGYEPAEVAVRVGKGVTLQNITLYPLSTTLYEPRSDPTLYR
CPA_CUPSA                RGEYWRILRPGTYTLQAFADGYEPAELTAHISNGVTMQNITLYPLSTTLYEPRSDPTLFR
CPA_ALOMA                KGEYWRILRPGTYTLQAFADGYAPAELTVHVSKGVTLQNVTLYPLSTTLYEPRSDPTLFR
                         :************.::. *:** *.*::  :.:***:**:**** ::* ****** *  *

TR_A0A087UBY6_STEMI      NSFPSTHNTASSIKGVWPTFTVVII----VIFNRISVALNVWKCGV--------------
CPA_VIRFA                SPG-----SRNTGAAVWPTITNLLKPKETVIFPRRRYVYRPFLAESTPNRLFTTNFDV--
CPA_CUPSA                PG-------HNTAAAVWPTLTNFLKPKETVIFPRRSYVYRPLVADPSTNRLFSTTTTRQL
CPA_ALOMA                AG-------PNSAAAVWPTLTNFLKPKETVIFPRRSYVYRPLVTEPATTNRILTT--IRY
                                   .:  .****:* .:     *** *   . .

TR_A0A087UBY6_STEMI      ------
CPA_VIRFA                ----YG
CPA_CUPSA                HYQILG
CPA_ALOMA                HHQLLG
```

Percent Identity Matrix - created by Clustal2.1

1:	tr_A0A087UBY6_STEMI	100.00	65.49	65.18	66.14
2:	CPA_VIRFA	65.49	100.00	79.01	74.73
3:	CPA_CUPSA	65.18	79.01	100.00	82.13
4:	CPA_ALOMA	66.14	74.73	82.13	100.00

Proteomic coverage trypsin

>CPA_CUPSA carboxypeptidase A-like protein

MFPSVLLLLL LCAGLSSC**VD VPFSYHDFSS MSRLMHLLHD ENPELTRIYS VGKSTQGRDL WVLLITHDPD SEPTLKPNVK**
YIANMHGNEA VGREMLLHLA SYLVSAYGSD DYVTWLLDHT RVHLMPSMNP DGFEASTEGQ CSGTQGRGNT NSFDLNRNFP
DYFTGDQDKL QPETRAVMR**W MAQTQFVLSA NLHGGALVAS YPFDNVPPNT PK**GFR**SSHSP SRTPDDDVFR HLAETYSFNH**
KSMYAGVSCR DGTPEFLNGT TNGAAWYPLK **GGMQDYNYVW AGCMEITLEI SCCKFPRAHR LPQFWEDNKH ALLKYLGEAH**
QGVWGVVSDS NNNPLNNVSI KVAGRQFGSR **TTHRGEYWRI LRPGTYTLQA FADGYEPAEL TAHISNGVTM QNITLYPLST**
TLYEPRSDPT LFRPGHNTAA AVWPTLTNFL KPKETVIFPR RSYVYRPLVA DPSTNRLFST TTTRQLHYQI LG

Proteomic coverage chymotrypsin

>CPA_CUPSA carboxypeptidase A-like protein

MFPSVLLLLL LCAGLSSC**VD VPFSYHDFSS MSRLM**HL**LHD ENPELTRIYS VGKSTQGRDL W**VL**LITHDPD SEPTLKPNVK**
YIANMHGNEA VGREMLLHLA SYLVSAYGSD DYVTW**LLDHT RVHLMPSMNP DGFEASTEGQ CSGTQGRGNT NSFDLNRNFP**
DYFTGDQDKL QPETRAVMRW MAQTQFVL**SA NLHGGALVAS YPFDNVPPNT PKGF**R**SSHSP SRTPDDDVFR HLAETYSFNH**
KSMYAGVSCR DGTPEFLNGT TNGAAWYPL**K GGMQDYNYVW** AGCMEITLEI SCCKFPRAHR LPQFWEDNKH ALL**KYLGEAH**
QGVWGVVSDS NNNPLNNVSI KVAGRQFGSR TTHRGEYWRI LRPGTYTLQA FADGYEPAEL TAHISNGVTM QNITLYPL**ST**
TLYEPRSDPT L**FRPGHNTAA AVWPTLTNFL KPKETVIFPR** RSY**VYRPLVA DPSTNRLF**ST TTTRQLHYQI LG

Supplementary Fig S1.5 Multiple sequence alignment of CPA from the venom glands of *C. salei* (CPA_CUPSA [MH766626/MH766627]), *A. marikovskyi* (CPA_ALOMA), *V. fasciatus* (CPA_VIRFA), and *S. mimosarum* (tr_A0A087UBY6_STEMI). Signal peptides are in black characters, cysteines are marked in white characters with a black box, positively charged amino acids in pink, negatively charged amino acids in blue, hydrophobic amino acids in red and polar amino acids in green. The Zn^{2+} binding site is highlighted in gray.
